# Supplementary material for: A nucleoid-associated protein is involved in the emergence of antibiotic resistance by promoting the frequent exchange of the replicative DNA polymerase in Mycobacterium smegmatis
Source: mSphere. 2024 Apr 9;9(5):e00122-24. doi: 10.1128/msphere.00122-24 (PMC11237743; doi:10.1128/msphere.00122-24)
Supplement: Supplemental material — Table S12 and Figures S1-S7. [file msphere.00122-24-s0003.pdf]

TABLES S1-6. All quantified RNA-seq read counts for WT and Δ*lsr2*, in triplicate, treated with mitomycin C at 0, 1, 3, 6, or 24 hours.
*see attached

TABLES S7-11. Differentially expressed genes in WT and Δ*lsr2* treated with mitomycin C 0, 1, 3, 6, or 24 hours
*see attached

TABLE S12. Strain list

STRAIN	GENOTYPE	SOURCE	Additional information
WN116	mc2155		wild-type strain
WN788	mc2155Δlsr2::zeoR	This work	*lsr2* deletion strain
WN906	mc2155Δlsr2::zeoR L5::plsr2-mScarletI-FLAG Giles::pimuAB-msfGFP-myc Tweety::pNative-lsr2	This work	*lsr2* deletion strain, complemented with untagged *M. smegmatis* Lsr2
WN874	mc2155Δlsr2::zeoR Giles::pimuAB-msfGFP-myc Tw::pdnaE2-mScarletI-FLAG L5::pNative-Rv3597c	This work	*lsr2* deletion strain, complemented with untagged *M. tuberculosis* Lsr2
WN1384	mc2155 Giles::pNative-dnae1-mScarletI-FLAG	This work	C-terminal DnaE1 fusion in a WT background
WN1385	mc2155Δlsr2::zeoR Giles::pNative-dnae1-mScarletI-FLAG	This work	C-terminal DnaE1 fusion in a Δ*lsr2* background
WN1390	mc2155Δlsr2::zeoR L5::pNative-lsr2-mScarletI-FLAG Giles::pTetO-DnaE1-mGFPmut3-myc	This work	Dual-labeled C-terminal Lsr2 and C-terminal DnaE1 fusions in a Δ*lsr2* background
WN573	mc2155ΔimuAB::loxP	This work	*imuA'B* deletion strain
WN1278	mc2155ΔimuAB::loxP L5::pJR962-LexA sgRNA Giles::pNative-imuA_FLAG-mScarletI-imuB-1550DS	This work	Translational fusion of ImuB
WN1280	mc2155ΔimuAB::loxP Δlsr2::zeoR L5::pJR962-LexA sgRNA Giles::pNative-imuA_FLAG-mScarletI-imuB-1550DS	This work	Translational fusion of ImuB in Δ*lsr2* background
WN1136	mc2155ΔimuAB::loxP Δlsr2::zeoR	This work	*imuA'B* and *lsr2* double knockout strain
WN855	mc2155 L5::plsr2-mScarletI-FLAG Giles::pimuAB-500-msfGFP-myc	This work	WT mc2155 with promoter fusions for *lsr2* and *imuA'B*
WN856	mc2155Δlsr2::zeoR L5::plsr2-mScarletI-FLAG Giles::pimuAB-500-msfGFP-myc	This work	Δ*lsr2* with promoter fusions for *lsr2* and *imuA'B*
WN858	mc2155Δlsr2::zeoR L5::pNative-lsr2-mScarletI-FLAG Giles::pimuAB-500-msfGFP-myc	This work	Δ*lsr2* with promoter fusions for *lsr2* and *imuA'B,* and the Lsr2-mScarletI-FLAG fluorescent translational fusion
WN1318	mc2155ΔimuAB::loxP L5::pJR962-LexA sgRNA Giles::pNative-imuA-mScarletI-FLAG_imuB-1550DS	This work	Translational fusion of ImuA'
WN273	mc2155Δdnae2::zeoR	This work	*dnaE2* deletion strain
WN171	mc2155 rpoB::mApple-hygR dnaE2::mGFPmut3-zeoR	This work	Translational fusion of DnaE2; endogenous *dnaE2* was fused to a fluorescent protein at the C-terminal

TABLE S13. Primer list

*see attached

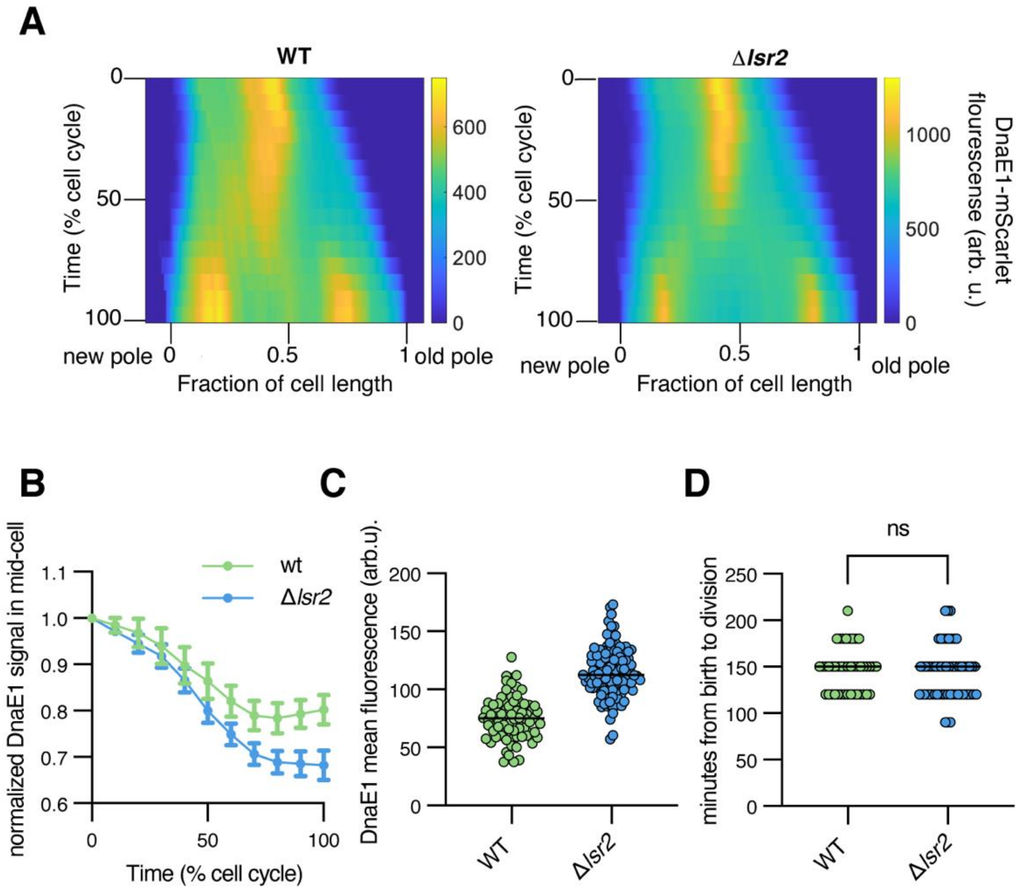

Figure S1. Loss of Lsr2 alters DnaE1 localization over the cell cycle. (A) Kymograph of DnaE1-mScarlet expressed from the native promoter on a phage integrative plasmid in WT (left, N=43) or Δ*lsr2* (right, N=59) (WN1384, WN1385). Kymograph for WT is the same as main text Figure 1B for comparison. **(B)** From the cells shown in panel A, the fluorescence at mid-cell from was normalized to time 0 and plotted over time. **(C)** Average DnaE1-mScarlet fluorescence in WT and Δ*lsr2* (N=73 for WT and 117 for Δ*lsr*2). **(D)** The time between birth and division, as determined by visible invaginations on the phase contrast images, was measured for both WT and Δ*lsr2* cells (N=43 for WT and 59 for Δ*lsr*2). Significance was determined using an unpaired t-test.

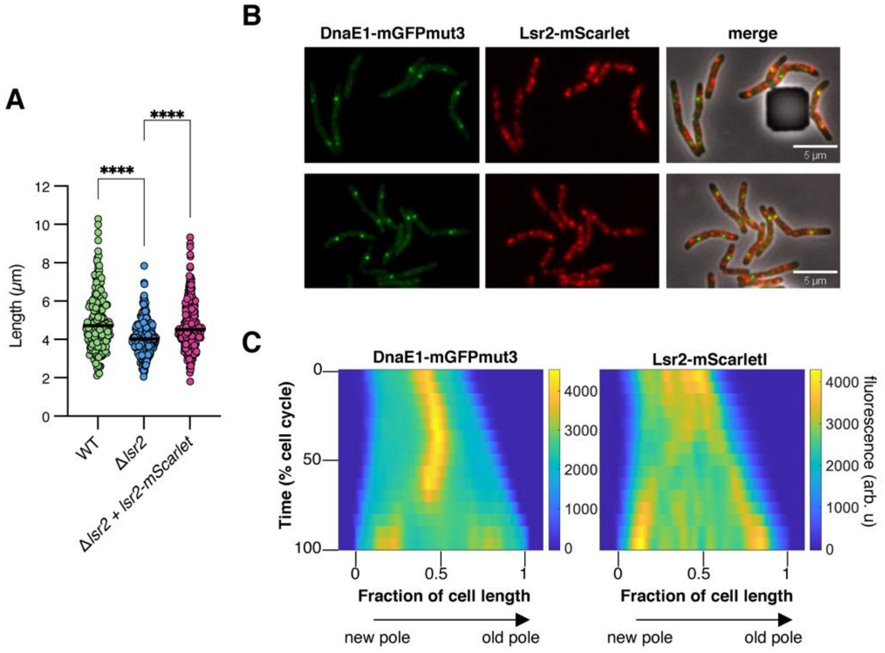

Figure S2. DnaE1 and Lsr2 do not co-localize. (A) Cell length of isogenic WT, Δ*lsr2,* and Δ*lsr2* expressing Lsr2-mScarlet from its native promoter (WN855, WN856, WN858; N=189, 326, and 291 cells, respectively). Dark lines represent medians of the single-cell distributions. p-values were obtained by one-way ANOVA. 0.1234 (ns), 0.0332 (*), 0.0021 (**), 0.0002 (***), <0.0001 (****) **(B)** Microscopy images of cells expressing DnaE1-mGFPmut3 and Lsr2-mScarlet in Δ*lsr2* (WN1390). **(C)** Average kymographs for each fusion (N=28 cells).

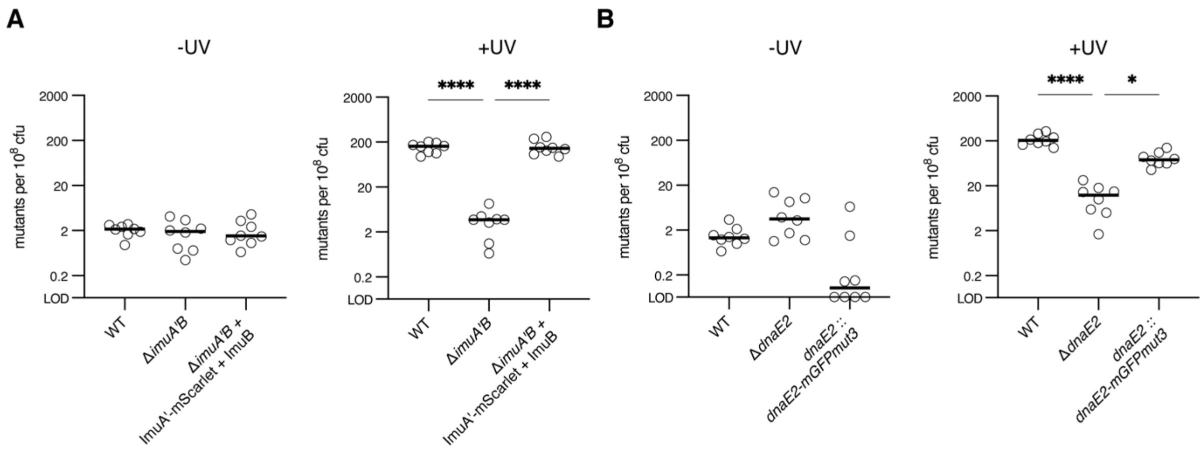

Figure S3. ImuA’ and DnaE2 translational fusions maintain functionality in UV-induced mutagenesis. Frequency of rifampin-resistant mutants per 10^8 cfu without or with UV-induced DNA damage (20 mJ/cm^2 UV). **(A)** Results shown are for WT, *imuA’B* deletion, or *imuA’B* deletion complemented with a fluorescent translational fusion of ImuA’ and ImuB (WN116, WN573, WN1278; 8 biological replicates). Complements are expressed from the native promoter on a phage integrative plasmid. **(B)** Results shown are for WT, *dnaE2* deletion, and a strain expressing a fluorescent translational fusion of DnaE2 from the native locus (WN116, WN273, WN171; 8 biological replicates). Middle horizontal line represents the median. LOD refers to the limit of detection. p-values were obtained by one-way ANOVA. 0.1234 (ns), 0.0332 (*), 0.0021 (**), 0.0002 (***), <0.0001 (****).

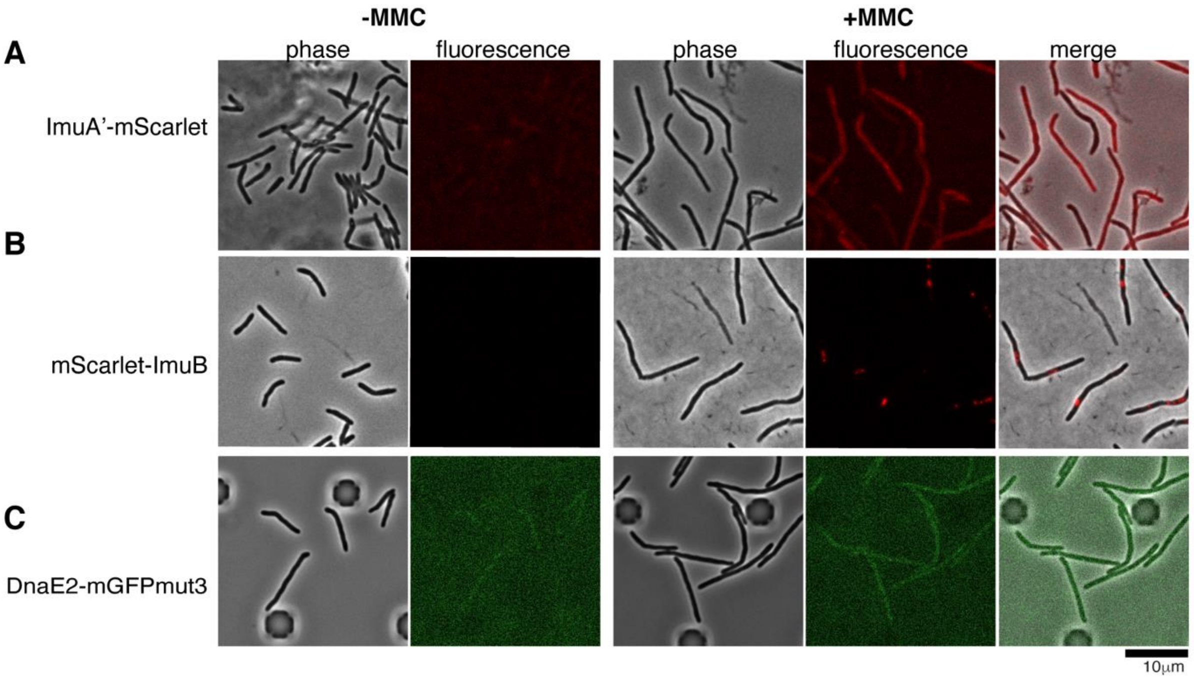

Figure S4. Microscopy images of mutasome translational fusions. (A) *imuA'B* deletion mutant complemented with a fluorescent translational fusion of ImuA' and untagged ImuB (WN1318). **(B)** *imuA'B* deletion mutant complemented with untagged ImuA' and a fluorescent translational fusion of ImuB (WN1278). Complements for panels A and B are expressed from the native promoter on a phage integrative plasmid. **(C)** A fluorescent translational fusion of DnaE2 was expressed from the native locus (WN171). DNA damage was induced by mitomycin C treatment (80 ng/ml) for 4-6 hours. Images were acquired using 1 second **(A, B)** or 200 ms **(C)** exposure times.

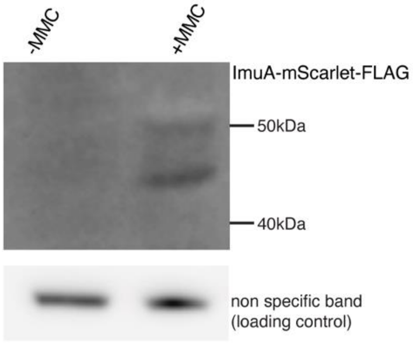

Figure S5. ImuA'-mScarlet-FLAG Western blot. Lysates from cells expressing ImuA'-mScarlet-FLAG were separated by SDS-PAGE and analyzed by anti-FLAG Western blotting. DNA damage was induced by mitomycin C treatment (80 ng/ml, 12 hours) (WN1318). A non-specific FLAG reactive band was used as the loading control.

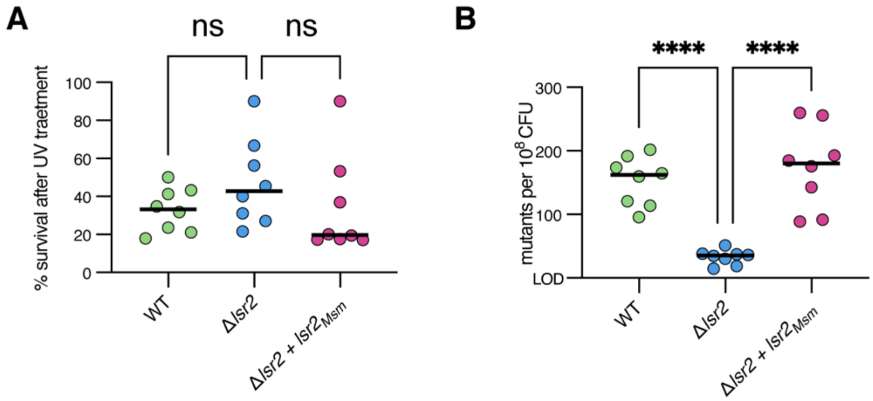

Figure S6. Survival and mutation following UV treatment. (A) WT, Δ*lsr2*, and complement (WN116, WN788, WN906; 8 biological triplicates), were treated with 20 mJ/cm^2 UV and plated for cfu. Percent survival is cfu/ml for UV-treated cells divided by the cfu/ml for untreated cells multiplied by 100. **(B)** Frequency of rifampin-resistant mutants per 10^8 CFU with UV-induced DNA damage (20 mJ/cm^2). *lsr2* complements were expressed from the native *lsr2* promoter on a phage integrative plasmid. Middle horizontal line represents the median. LOD refers to the limit of detection. p-values were obtained by one-way ANOVA (****), p<0.0001. 0.1234 (ns), 0.0332 (*), 0.0021 (**), 0.0002 (***), <0.0001 (****).

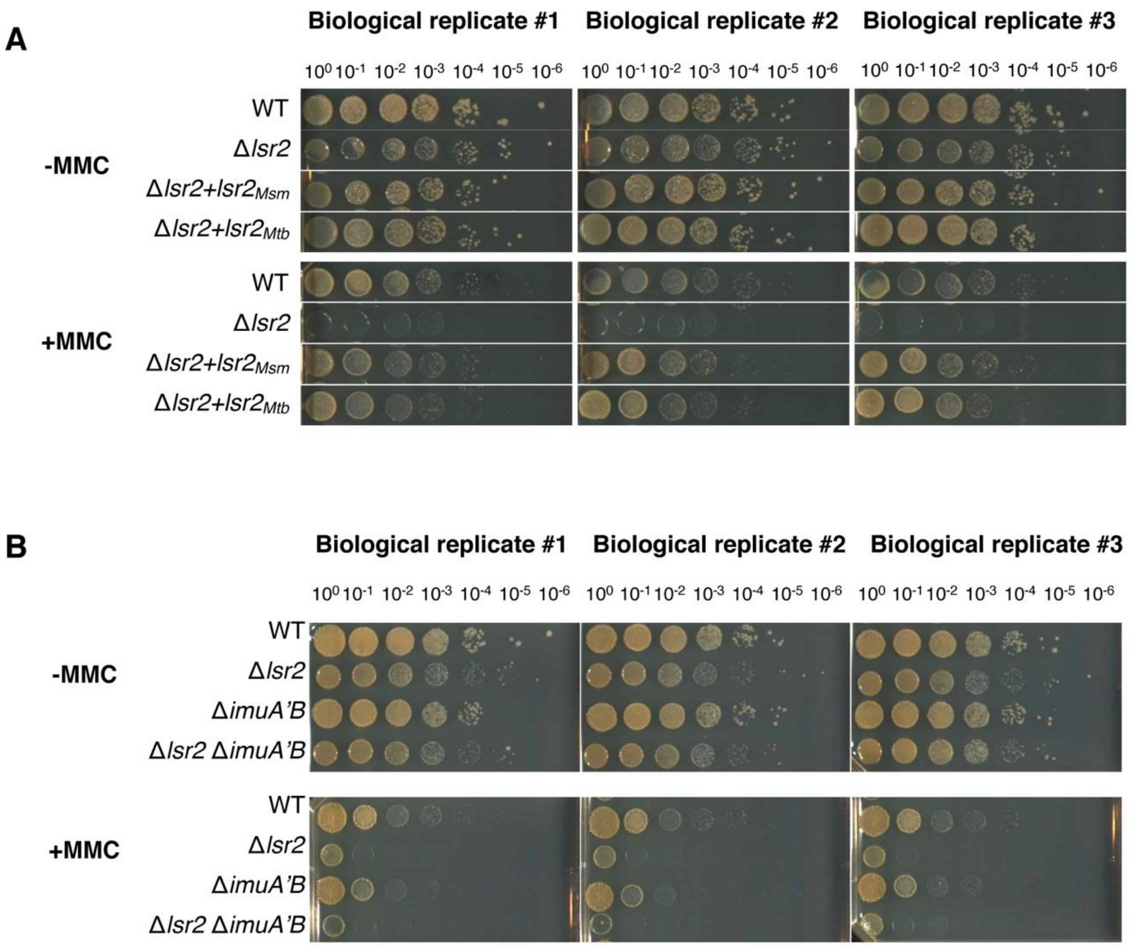

Figure S7. Loss of *lsr2* leads to growth defects on mitomycin C. (A) Serial dilutions of WT, Δ*lsr2*, and complemented strains were spotted on agar plates with or without mitomycin C (1.56 ng/ml) in biological triplicate (WN116, WN788, WN906, WN874). *M. smegmatis* and *M. tuberculosis lsr2* complements were expressed from the native *lsr2* promoter on a phage integrative plasmid. **(B)** Serial dilutions of WT, Δ*lsr2*, Δ*imuA'B,* and Δ*lsr2* Δ*imuA'B* strains were spotted on agar plates with or without mitomycin C (1.56 ng/ml) in biological triplicate.
